# PAK1-mediated MORC2 phosphorylation promotes gastric tumorigenesis

**DOI:** 10.18632/oncotarget.3185

**Published:** 2015-03-21

**Authors:** Guiling Wang, Yanyan Song, Tong Liu, Chunyu Wang, Qing Zhang, Furong Liu, Xinze Cai, Zhifeng Miao, Hongde Xu, Huimian Xu, Liu Cao, Feng Li

**Affiliations:** ^1^ Department of Cell Biology, Key Laboratory of Cell Biology, Ministry of Public Health, and Key Laboratory of Medical Cell Biology, Ministry of Education, China Medical University, Shenyang, China; ^2^ Department of Surgical Oncology and General Surgery, First Hospital of China Medical University, Shenyang, China

**Keywords:** MORC2, phosphorylation, P21-activated kinase 1(PAK1), gastric cancer

## Abstract

To date, microrchidia (MORC) family CW-type zinc-finger 2 (MORC2), has been found to be involved in p21-activated kinase1 (PAK1) pathway to maintain genomic integrity. Here, we explore its novel role in cancer. We demonstrate that PAK1-mediated MORC2 phosphorylation promotes cell cycle progression, defective phosphorylation of MORC2-S677A results in attenuated cell proliferation and tumorigenicity of gastric cancer cells, which is significantly enhanced in overexpression of phospho-mimic MORC2-S677E form, suggesting the importance of MORC2 phosphorylation in tumorigenesis. More importantly, phosphorylation of MORC2 correlates positively with PAK1 expression in clinical gastric cancer. Furthermore, high expression of PAK1 and phosphorylation of MORC2 appear to be associated with poor prognosis of clinical gastric cancer. Collectively, these findings revealed a novel function of MORC2 phosphorylation in promoting gastric cell proliferation *in vitro* and tumorigenesis *in vivo*, suggesting that blocking PAK1-mediated MORC2 phosphorylation might be a potential therapeutic strategy for gastric tumorigenesis.

## INTRODUCTION

Gastric cancer is the second-third most common cause of cancer-related death in the world [1]. As with other cancers, the development of gastric cancer is a multistep process. Deregulated cell proliferation is a key mechanism for tumor progression [2]. To improve cancer patient survival, it is a central event to investigate the proteins governing development and progression of gastric cancer.

The PAK1 is a serine/threonine protein kinase which is stimulated by active Rac1 and Cdc42-GTPases [3–5], and has been found to be key regulator of cancer-cell signaling networks [6]. There has been mounting evidence that PAK1 is tightly related to the progression of cancer and may become a promising diagnostic and therapeutic target for cancer [7–12]. Therefore, it is worthwhile to study the novel binding partners of PAK1. Here, we indicated that PAK1 phosphorylates MORC2 at Ser677, which is the same as has been previously reported (Ser739) [13], but the role of MORC2 phosphorylation is different.

Human MORC2 (microrchidia family CW-type zinc-finger 2), also known as KIAA0852, ZCW3 or ZCWCC1, containing a CW-type zinc-finger and three coiled-coil domains [14], is a member of the MORC protein family and mainly localizes in the nucleus [15]. Human MORC proteins have been classified into two subfamilies: subfamily I including MORC1 and MORC2, while MORC3 and MORC4 belong to subfamily IX [14]. To date, microrchidia (MORC) family CW-type zinc-finger 2 (MORC2), has been found to be involved in several functions including regulation chromatin remodeling during the DNA-damage response [13], repression gene transcription [15, 16], promotion lipogenesis by regulating ACLY activity [17].

The function of MORC2 in gastric cancer has not yet been investigated. In this study, we showed that phosphorylation of MORC2 by PAK1 promotes gastric cell proliferation *in vitro* and tumorigenesis *in vivo*. There was a strong positive correlation between PAK1 and phosphorylation of MORC2 expression in gastric cancer samples. This report is the first investigation focused on exploring the role of MORC2 phosphorylation in gastric cancer.

## RESULTS

### PAK1 phosphorylates MORC2 at Ser-677 *in vivo*

In our study, we also found that PAK1 interacts with MORC2 (Supplementary Figure S1a and S1b) and phosphorylats MORC2 at Ser-677 *in vitro* (Supplementary Figure S1c and S1d) While our work was in progress, a published paper showed that the identified MORC2 phosphorylation site at Ser739 [13], which is the same as ours' finding (Ser677). The difference was resulted from using different MORC2 reference sequences from the GenBank. Here we have explored the novel function of this phosphorylation site in gastric cancer.

**Figure 1:**
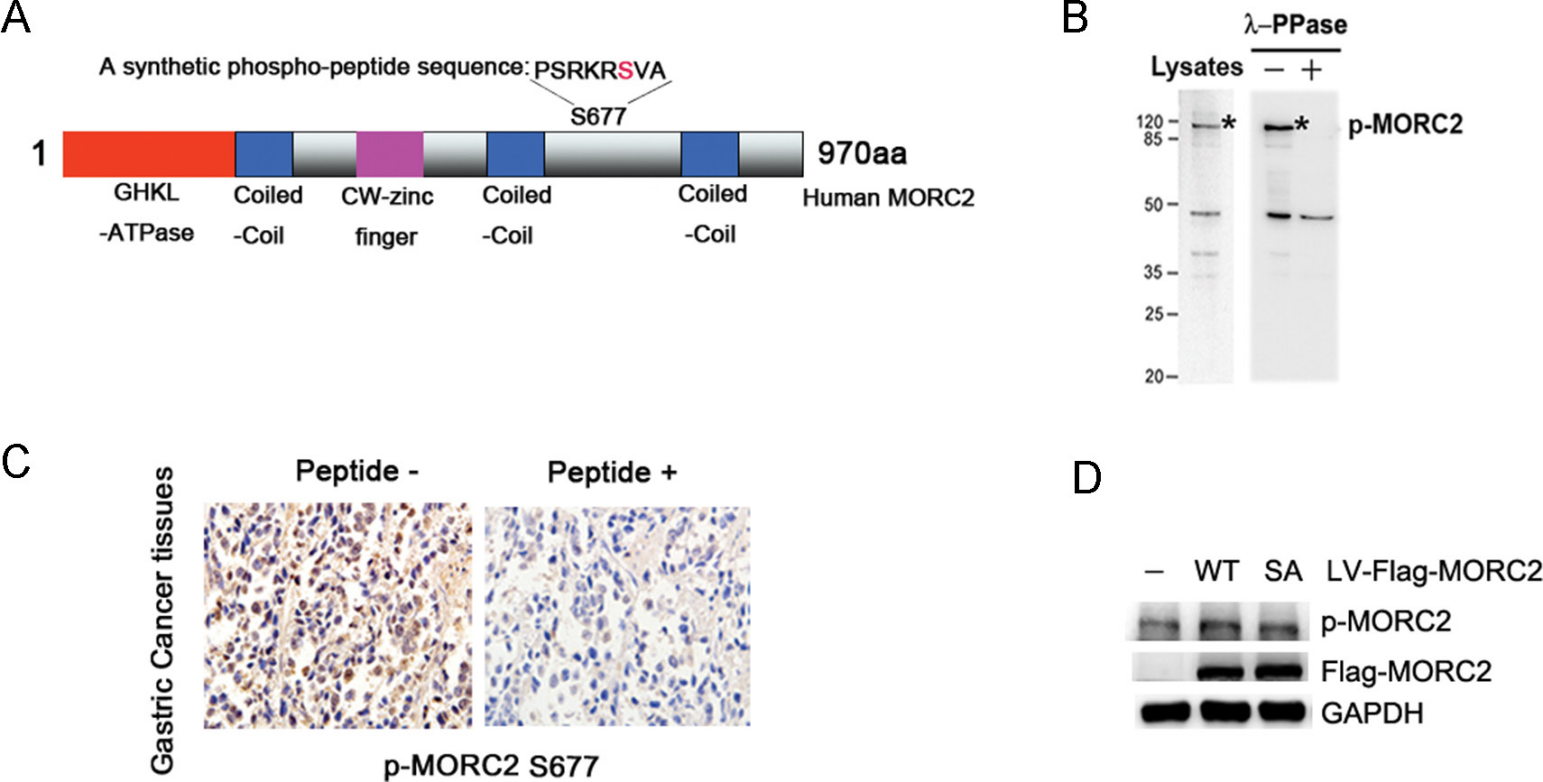
PAK1 phosphorylates MORC2 at Ser-677 in intact cells **(A)** The p-MORC2 antibody was produced by immunizing animals with a synthetic phospho-peptide (−PSRKRSVA) sequence corresponding to residues surrounding Ser677 of human MORC2 (NM-014941.1). Potential phosphorylation site of human MORC2 (S677) is shown in red. **(B)** Detection of endogenous MORC2 phosphorylation using phospho-MORC2 S677 antibody by western blot assays when cells were treated with and without λ-PPase. **(C)** Detection of MORC2 phosphorylation using phospho-MORC2 S677 antibody by immunohistochemistry assays in gastric cancer tissues. Left, paraffin section of gastric cancer tissues were used in immunohistochemistry and detected by anti-p-MORC2 S677 (5 μg/ml). Right, for peptide competition expreiments, after incubation with antigenic phospho-peptide (100 μg/ml), phospho-MORC2 S677 antibody (5 μg/ml) was used for immunohistochemistry in paraffin section from the same case. **(D)** The levels of MORC2 phosphorylation were detected using phospho-MORC2 S677 antibody by Western blotting in the lentivirus-mediated stable expressing of Flag-MORC2-WT/SA SGC-7901 cells.

To determine the role of MORC2 phosphorylation at serine 677 *in vivo*, we used a phosphorylation state-specific antibody raised against phospho-MORC2 Ser677 to confirm the phosphorylation of MORC2 *in vivo* (Figure 1A). The specificity and reactivity of the antibody were verified with or without λ-PPase in BGC-823 cells (an endogenous MORC2 relatively high expression gastric cancer cell line, see Figure 1B) and gastric cancer tissues (Figure 1C). Next to determine whether the MORC2 phosphorylation at Ser677 mutant affects its phosphorylation using the phospho-MORC2 Ser677 specific antibody, we constructed the stable expressing of wild-type MORC2 (MORC2-WT), nonphosphorylatable MORC2 S677A mutant (MORC2-SA), phospho-mimicking MORC2 S677E mutant (MORC2-SE) and Flag-vector control in SGC-7901 cell lines. Western blot results indicated that MORC2 S677A mutation attenuated the phosphorylation of MORC2 on serine 677 in Flag-MORC2/SGC-7901 cells (an exogenous MORC2 stable expression gastric cancer cell line, see Figure 1D) compared with wild-type MORC2 (MORC2-WT). These results indicated that PAK1 can phosphorylate MORC2 at Ser677 in intact cells

### Phosphorylation of MORC2 at Ser677 is dependent on PAK1

**Figure 2:**
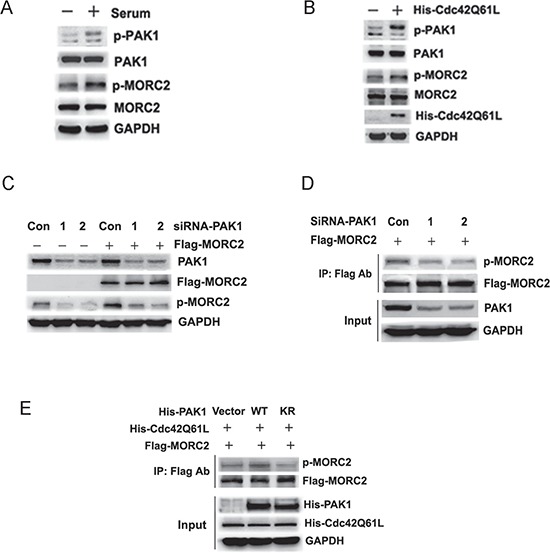
MORC2 phosphorylation at Ser-677 is dependent on PAK1 **(A)** Serum treatment resulted in an increase in phosphorylation levels of endogenous MORC2 in BGC-823 cells via PAK1. BGC-823 cells were starved over night and stimulated with 10% (v/v) serum for 45 min to activate PAK1. The lysates were probed with indicated antibodies. **(B)** Activated Cdc42 resulted in up-regulation of MORC2 phosphorylation at Ser 677 via PAK1. BGC-823 cells were transfected with His-Cdc42Q61L and performed by Western blot. The lysates were probed with indicated antibodies. MORC2 phosphorylation at Ser677 was blotted with MORC2 phospho-specific antibody. **(C)** Western blot analysis of the level of MORC2 phosphorylation by blocking the upstream PAK1 expression with two different siRNAs (#1 and #2) targeting PAK1 when MORC2 was over-expressed. The lysates were probed with indicated antibodies. **(D)** The effect of PAK1 on MORC2 phosphorylation with ectopic MORC2 by Flag IP assays when endogenous PAK1 was knocked down. The western blot analysis was probed with indicated antibodies. **(E)** The effect of PAK1 on MORC2 phosphorylation with ectopic MORC2 by Flag IP assays when PAK1 was overexpressed in the presence of Cdc42 (Cdc42Q61L). The western blot analysis was probed with indicated antibodies.

Previous studies have shown that serum activates PAK1 [18], we next determined whether serum treatment could induce MORC2 phosphorylation by PAK1 kinase. In these experiments MORC2 phosphorylation at Ser677 was assayed by western blotting using the phospho-MORC2 Ser677 specific antibody. Our results showed that serum treatment resulted in an increase in phosphorylation levels of endogenous PAK1 and MORC2 Ser 677 in BGC-823 cells (Figure 2A), suggesting that MORC2 phosphorylation may be induced by serum in a PAK1 kinase-dependent manner. Given that PAK1 was an effector of activated Cdc42 [19], we investigated whether PAK1-mediated MORC2 phosphorylation was downstream of activated Cdc42. The results showed that activated PAK1 further facilitated MORC2 phosphorylation on serine 677 in the presence of Cdc42 (Cdc42Q61L) (Figure 2B), which suggest that activated Cdc42 promotes up-regulation of MORC2 phosphorylation at Ser677 via PAK1.

To further demonstrate the importance of PAK1 in MORC2 phosphorylation at Ser677 in cultured cells, endogenous PAK1 was knocked down by two different siRNAs (#1 and #2) targeting PAK1. The efficacy of PAK1 siRNA was demonstrated by depletion of PAK1 (Figure 2C, first panel). In these experiments MORC2 phosphorylation at Ser677 was performed by western blotting using the phospho-MORC2 Ser677 specific antibody. Compared with control siRNA, there is a decrease in MORC2 phosphorylation with endogenous or ectopic MORC2 expression in these cells when PAK1 was knocked down (Figure 2C). Besides, the Flag IP results further indicated that depletion of the endogenous PAK1 resulted in a reduction of MORC2 phosphorylation with ectopic MORC2 (Figure 2D). The wild-type PAK1 (PAK1-WT) promoted the phosphorylation of MORC2 at Ser677 compared with kinase-dead PAK1(PAK1-KR) in the presence of Cdc42 (Cdc42Q61L) (Figure 2E), suggesting that activated PAK1 promotes up-regulation of MORC2 phosphorylation at Ser677. Taken together, these results clearly showed that PAK1 affected MORC2 phosphorylation at Ser677, which suggests that MORC2 phosphorylation at Ser677 is dependent on PAK1.

### Phosphorylation of MORC2 promotes gastric cancer cell proliferation

**Figure 3:**
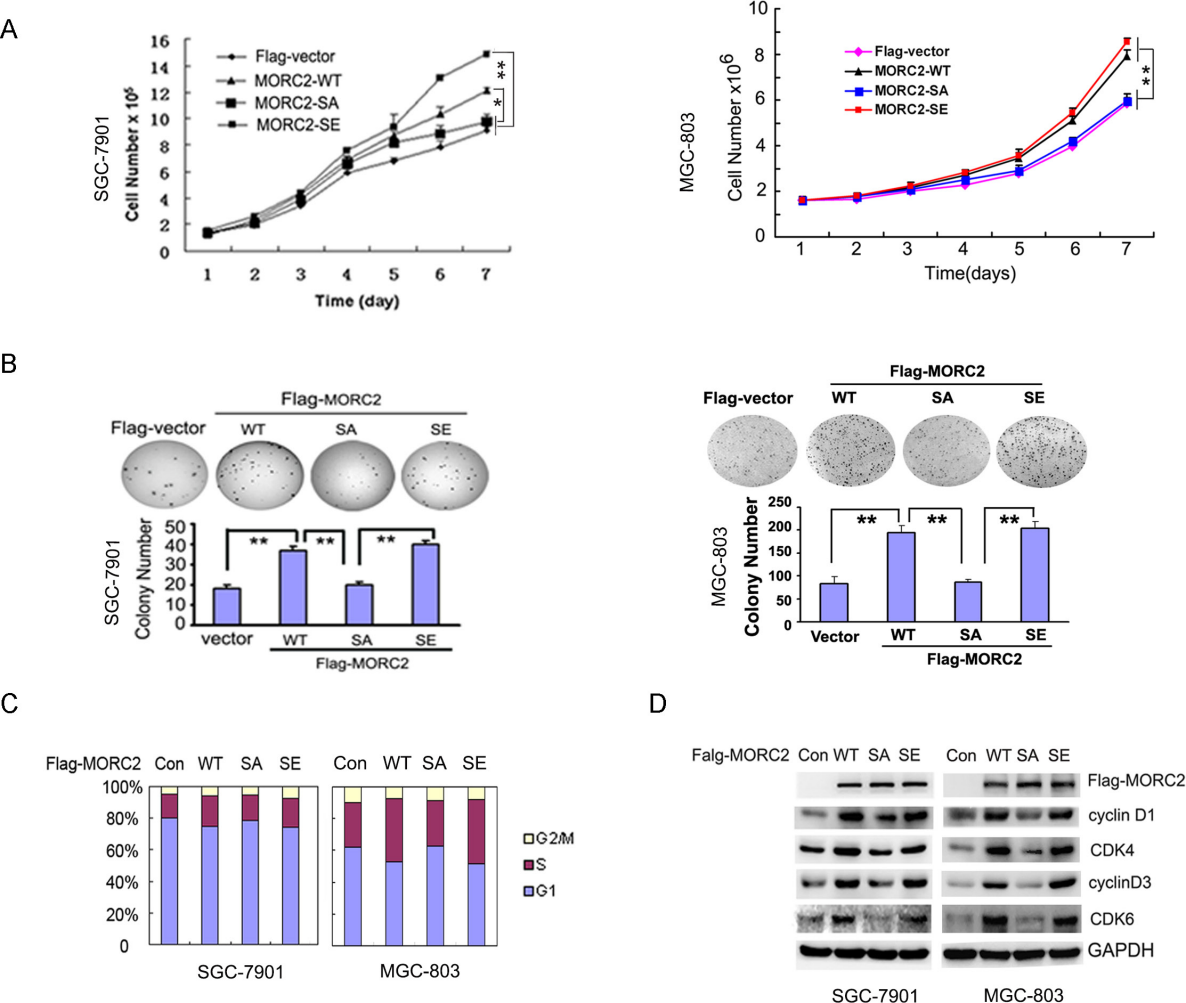
Phosphorylation of MORC2 at Ser-677 promotes gastric cell proliferation and cell cycle progression **(A and B)** MORC2 Phosphorylation promotes gastric cell proliferation. (A) The stable expressing lentivirus-mediated Flag-MORC2 SGC-7901 and MGC-803 cells grown for 2,4 or 6 days and analyzed for cell growth by cell counts assay. Values are the means ± SD from three individual experiments. (B) Colony formation assays were performed with the lentivirus-mediated stable expressing of Flag-MORC2 SGC-7901 and MGC-803 cells. Representative results are shown. **(C–D)** Phosphorylation of MORC2 promotes cells transition from G1 to S. (C) The lentivirus-mediated stable expressing of Flag-MORC2 of SGC-7901 and MGC-803 cells were cultured and stained with propidum iodide. Cell cycle distribution was measured by FACS. Values are the means ± SD from three individual experiments. The average percents of three experiments were showed in histogram. (D) The levels of these proteins controlling cell cycle were detected by Western blotting in the lentivirus-mediated stable expressing of Flag-MORC2 SGC-7901 and MGC-803 cells. The lysates were probed with indicated antibodies.

In our study, we observed this phenomenon that the stable expressing of MORC2-WT and MORC2-SE cells grow faster than MORC2-SA and vector control cells. To further confirm the notion, we carried out growth curves and colony formation assays with these cells. As shown in Figure 3A, MORC2-WT and MORC2-SE promoted SGC-7901 and MGC-803 cell growth to a greater extent than vector control and MORC2-SA. In addition, there was significant evidence to show that MORC2-WT and MORC2-SE formed more colonies *in vitro* relative to that seen with MORC2-SA and vector control (Figure 3B). These findings suggest that MORC2 phosphorylation may facilitate the growth and proliferation of gastric cancer cells.

### Defective phosphorylation of MORC2 results in attenuated cell cycle transition from G1 to S

To further investigate the role of MORC2 phosphorylation and explore the underlying mechanism in cancer cells, we performed flow cytometry analysis using these stable expressing MORC2 (WT, SA and SE) SGC-7901 cells. MORC2-WT and MORC2-SE cells showed significant decrease (MORC2-WT cells from 82.11% to 47.28% in average, MORC2-S677E cells from 83.39% to 39.33% in average) in the percentage of G1 phase cells, and significant increase in the percentage of S (MORC2-WT cells from 8.73% to 35.11% in average, MORC2-S677E cells from 5.68% to 37.07% in average) compared with vector control (from 91.32% to 75.65% in G1 average; from 3.43% to 16.43% in S average) and MORC2-SA cells (from 82.62% to 69.17% in G1 average; from 10.78% to 22.61% in S average), the cell cycle transition in MORC2-SA cells is not significant (Figure 3C, column 2 and column 4 compared to column 3 and column 1) compared with vector control. We also found that there was no significant change in the percentage of G2/M phase cells in MORC2-WT (from 9.14% to 17.6% in average) and MORC2-SE (from 10.92% to 19.57% in average) compared with MORC2-SA (from 6.59% to 8.21% in average) and vector control (from 5.25% to 8.01% in average). In addition, the similar results were also shown in the stable expressing MORC2 MGC-803 cells. Thus, the results indicate that phosphorylation of MORC2 mainly promotes gastric cancer cell cycle transition from G1 to S.

To confirm this observation, we decided to detect the levels of these proteins of cell cycle with these stable expressing gastric cancer cells. Compared to MORC2-SA and vector control, MORC2-WT and MORC2-SE dramatically increased the levels of cyclinD1/CDK4 and cyclinD3/CDK6 (Figure 3D). Taken together, the result indicated that the expression level of these proteins controlling cell cycle by western blotting are correlated with that by flow cytometry analysis, suggesting that MORC2 phosphorylation promotes cell cycle transition from G1 to S.

### Phospho-mimicking MORC2 enhances tumorigenesis of gastric cancer cells

To examine the effect of phosphorylated MORC2 on tumorigenicity of gastric cancer cells *in vivo*. SGC-7901 cells with stable expressing of vector control, MORC2-WT MORC2-SA and MORC2-SE were injected into nude mice via right scapular region. Four weeks after injection, the xenografts tumor was obtained for microscopic histological analysis.

**Figure 4:**
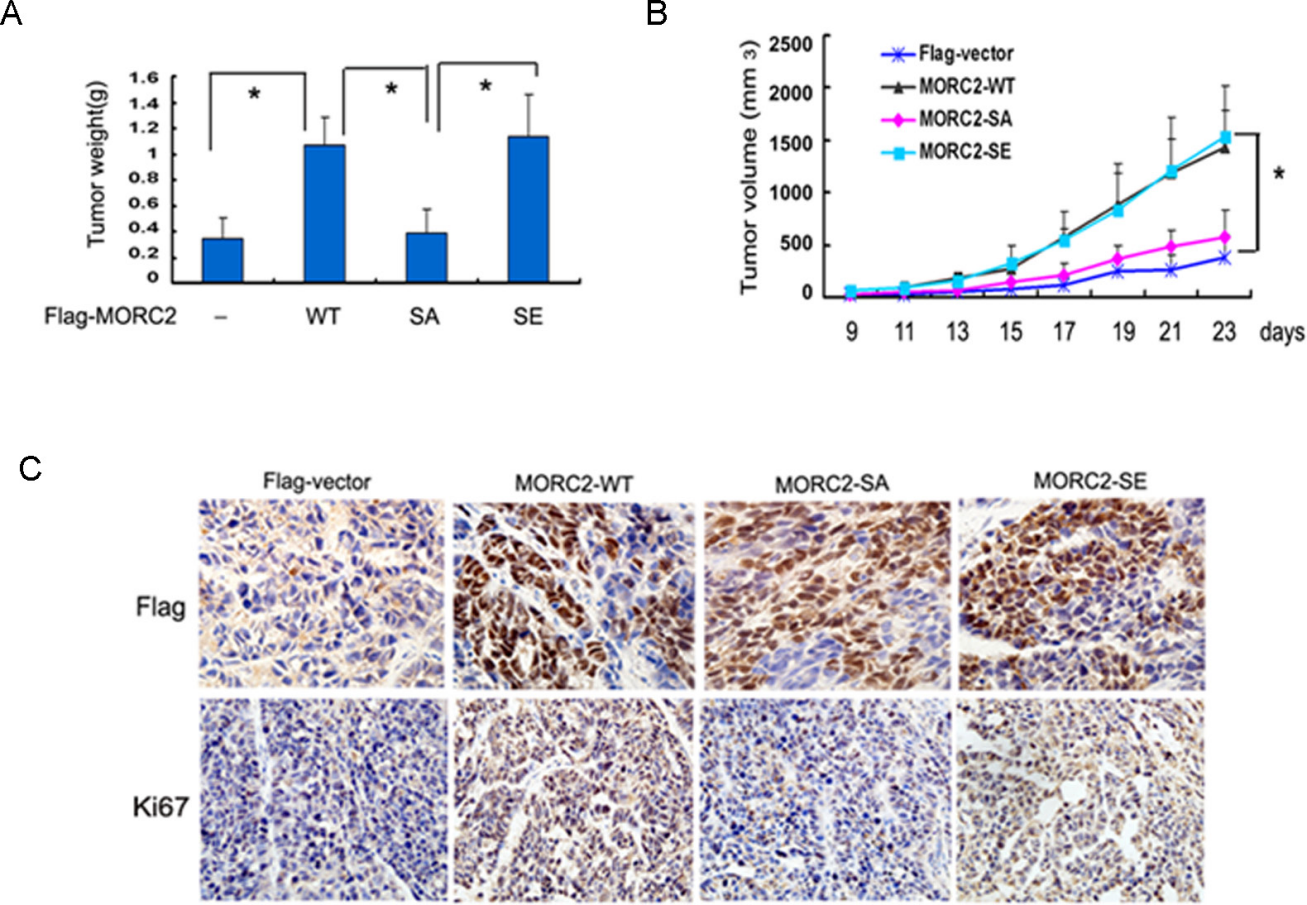
Defective phosphorylation of MORC2 at Ser-677 results in attenuated tumorigenesis in gastric cancer cells **(A)** The stable expressing lentivirus-mediated Flag-MORC2 SGC-7901 cells were injected into right scapular region of nude mice (each group = 4). The inoculated mice were terminated in 3 weeks. Each tumor lump was removed from the body. Photograghs of tumor weight was quantified. **(B)** Tumor volumes were measured weekly and data are mean ± SEM. **(C)** Immunohistochemistry staining of anti-Flag in nude mice tumor tissues sections, Original magnification, ×400. Degree of intratumoral proliferation was determined by Ki67 staining.

As shown in Figure 4A and 4B, nude mice injected with MORC2-WT or MORC2-SE SGC-7901 cells developed markedly heavier tumor weight and larger volume than vector control and MORC2-SA group. After that, we performed immunohistochemical analysis to verify the formation of xenografts tumor with anti-Flag staining in nude mice (Figure 4C, upper panel). Concurrently, accelerated proliferation was revealed Ki67 staining performed in xenografts tumor sections (Figure 4C, down panel). Taken together, MORC2 phosphorylation contributes to tumorigenesis of gastric cancer cells.

### PAK1 expression and phosphorylation of MORC2 positively correlates with gastric cancer clinical outcome

**Figure 5:**
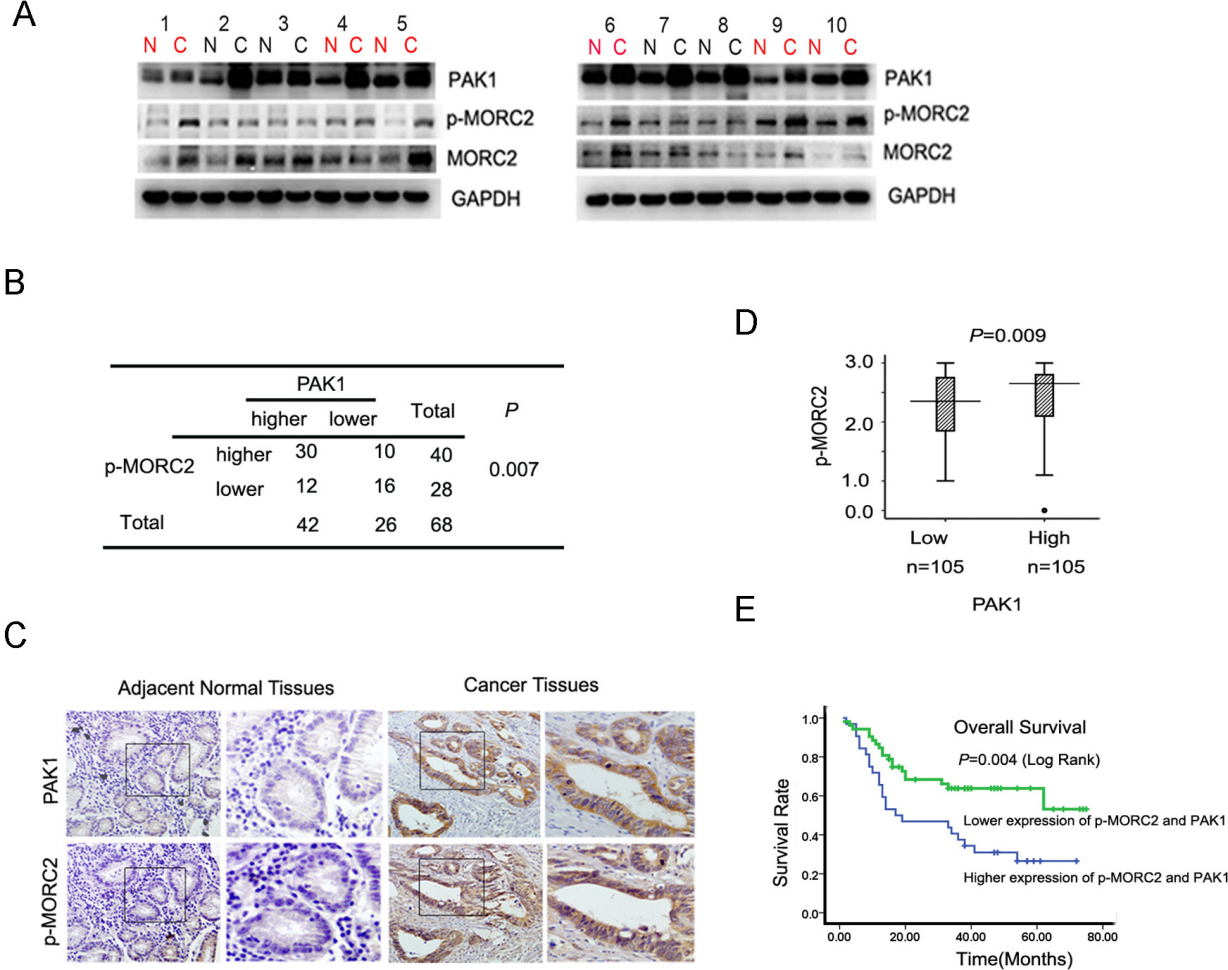
Phosphorylation of MORC2 and PAK1 expression positively correlates with clinical gastric cancer outcome **(A)** Evaluate the expression of the PAK1 and p-MORC2 in clinical tissues by western Blot. Lysates of 68 cancer tissues (C) and matched adjacent normal tissues (N) pairs were analyzed by Western Blot. Number of cancers with reduced or increased levels of indicated protein relative to normal adjacent tissues and analyze with GAPDH as the reference. The representative 10 pairs were shown. **(B)** Summary of the expression in tissues in (a) is shown, with tissues categorized by lower and higher expression. The expression of PAK1 and p-MORC2 Ser677 was analyzed with GAPDH as the reference. In each N and C pair, the lower/higher expression in C, compared with N, is categorized as lower/higher expression. The P value was generated using the chi-square test. **(C)** Representative images of immunohistochemical staining of PAK1 and p-MORC2 expression from one case were shown. The boxed areas in the left images are magnified in the right images. N, adjacent normal tissue (shown in the left column); C, cancer tissue (shown in the right column). Original magnification, ×200. **(D)** Box plot of PAK1 and p-MORC2 1 expressions were shown. The subjects were divided into two groups based on PAK1 expression scores in the 210 gastric tumors, representing low and high expression. The PAK1 and p-MORC2 expression scores were shown as box plots, with the horizontal lines representing the median; the bottom and top of the boxes representing the 25th and 75th percentiles, respectively; and the vertical bars representing the range of data. And extreme cases were marked with a dot. Data was analyzed by one-way analysis of variance (ANOVA) test with Games–Howell's correction. **(E)** Kaplan–Meier's analyses illustrated that the 5-year survival rate according to PAK1 and p-MORC2 expression scores, representing low and high expression of them. These 210 gastric cancer patients were divided into four groups based on PAK1 and p-MORC2 expression scores, the higher and lower of expression of PAK1 and p-MORC2 were analyzed by Kaplan–Meier analysis.

To further study the role of MORC2 phosphorylation in gastric cancer, we analyzed levels of PAK1and phosphorylated MORC2, by western blotting, in 68 pairs of gastric cancers and matched adjacent normal gastric tissue samples. The results incidated that overexpression of both p-MORC2 and PAK1 was observed in 44% (30 of 68) of gastric cancer, and neither of these two proteins presented higher expression in 23.5% (16 of 68) of tumors. MORC2 was phosphorylated in 71% (30 of 42) of gastric cancer with overexpressed PAK1 patients (Figure 5A). Statistical analysis revealed p-MORC2 expression positively correlated with PAK1 expression (*p* = 0.007; Figure 5B) in gastric cancer tissues, which are consistent with our findings that overexpression of PAK1 upregulated p-MORC2 in gastric cell lines (Figure 2).

After that, immunohistochemical staining for PAK1 and p-MORC2 was also performed using sequential sections from the same tissue, which indicated that the expression level of p-MORC2 by immunohistochemistry are correlated with that by western blotting. Representative samples were shown in Figure 5C. To better understand the correlation between them, we divided tumor samples into two groups on the basis of PAK1 amounts (cut off at the median score), and studied the differences of p-MORC2 expression with clinical gastric cancer. The data showed the expression scores of p-MORC2 in tumors were consistent with PAK1 scores ( *p* = 0.009; Figure 5D), and the high expression scores of PAK1 and phosphorylated MORC2 in clinical gastric cancer were also found to predict shorter overall survival of patients Log Rank *p* = 0.004, Figure 5E), suggesting that phosphorylation of MORC2 appears to be associated with the poor prognosis of clinical gastric cancer.

## DISCUSSION

Emerging data suggest that PAK1 is overexpressed in human cancer [7, 8], including gastric cancer [9–11]. The oncogenic activity of PAK1 is quite certain. However, the diverse signaling pathways are complicated, with accumulation and alteration of enormous genetic and epigenetic genes deregulated [20, 21]. Here, we demonstrate that MORC2 phosphorylation is involved in PAK1-mediated gastric tumorigenesis. These findings also identify a new way that PAK1-mediated MORC2 phosphorylation promotes tumorigenesis.

In this study, we used a phosphorylation state-specific antibody raised against phospho-MORC2 Ser677 to confirm the phosphorylation of MORC2 *in vivo*. The phospho-MORC2 Ser677 antibody was produced by immunizing animals with a synthetic phospho-peptide corresponding to residues surrounding Ser677 of human MORC2 (NM-014941.1), which had not share the identical (−PSRKRSVA) sequences with other MORC protein family. The specificity and reactivity of the antibody were verified by a series of assays. Thus, our findings provide important evidence to show the role of MORC2 phosphorylation in gastric cancer *in vivo*.

In our previous study, we found that MORC2 may act as a transcriptional repressor and play a role in cancer [15, 16], which promoted us to identify the underlying mechanism in cancer. Deregulated cell proliferation is a key mechanism for tumorigenesis, among which controlling cell cycle progression at the G1-S and G2/M has been considered as key step of cell proliferation and survival through regulating the activity of several cyclin-CDK complexes [22, 23]. Recent study indicated that PAK1–MORC2 is critical for orchestrating the interplay between chromatin dynamics and the maintenance of genomic integrity [13]. But our findings provide evidence that phosphorylation of MORC2 increases these proteins expression of cyclinD1-CDK4 and cyclinD3-CDK6 complexes, which promotes gastric cell cycle transition from G1 to S. Furthermore, phosphorylation of MORC2 leads to an increase in proliferation in gastric cancer cells both *in vitro* and *in vivo*, indicating that excessive activation of MORC2 may be critical for tumorigenicity of gastric cancer cells. In addition, our data presented here show that high expression of phosphorylated MORC2 and PAK1 correlates postively with clinical poor prognosis of gastric cancer. Therefore, we here, established, for the first time, that there was a relationship between PAK1 and MORC2 in gastric cancer.

However, it is possible that phosphorylation of MORC2 by PAK1 also affects other cellular processes. For example, it may also influence gastric cancer cell invasion and metastasis which will be studied in our future.

In summary, we report that the potential function of the PAK1-MORC2 axis in gastric tumorigenesis. Therefore, our data suggest that the MORC2 phosphorylation may play a critical role in the development of cancer, which may provide an important insight into understand the mechanisms of tumorigenesis and have significant therapeutic implications.

## MATERIALS AND METHODS

### Plasmid construction and mutagenesis

PAK1 expression plasmid was provided by Dr. Chernoff J [8]. His-Cdc42Q61L was a gift from Dr. Bokoch GM. pCDNA3.1-MORC2 (His-MORC2-WT) was used previously in our paper [15]. GST-tagged PAK1 or MORC2 and Flag-tagged MORC2 (MORC2-WT), nonphosphorylatable MORC2-S677 (MORC2-SA) and phospho-mimicking MORC2-S677 (MORC2-SE) were constructed by PCR amplification and subcloned into p3 × Flag CMV (Sigma) and pGEX-5x-1 (GE Healthcare) vectors, respectively, using His-MORC2-WT plasmid as a template. Mutations on MORC2 were generated by site-directed mutagenesis by the Quickchange-XL Site-Directed Mutagenesis kit (Stratgene).

### Cells culture, gene transfection and western blot

Human gastric cancer cell lines were cultured in DMEM (Invitrogen) supplemented with 10% fetal calf serum (Invitrogen), 100 units/ml penicillin and 100 μg/ml streptomycin at 37°C in a humidified atmosphere of 5% CO2. Cells were transfected with siRNA and plasmid vectors using Lipofectamine 2000 (Invitrogen). Western blot used in this study have been described previously in detail [24, 25].

### Antibodies

The following antibodies were used in the experiments: PAK1 and p-PAK1 (Cell Signaling), MORC2 (Bethyl Laboratories); phospho-MORC2 S677 (Bioss Inc); cyclinD1, cyclinD3, CDK4 and CDK6 (Cell Signaling); Ki67 (Santa Cruz); Flag-tagged Ab (Sigma); His-tagged Ab (GenScript Corporation); GAPDH (Kangchen, Shanghai). λ-PPase (New England Biolabs).

### SiRNA and lentiviral production

SiRNA-PAK1 and control siRNA were purchased from Nanjing genepharma Company. The siRNA PAK1 #1 sequence was 5′-AGAGCU GCUACAGCAUCAA-3′; the siRNA PAK1 #2 sequence was 5′-CUCCAAACCCAGAGGAGAAGA-3′ and the siRNA control sequence was 5′UUCU CCGAACGUGUCACGU-3′. pGC-Flag-vector-Lentivirus and pGC-Flag-MORC2-Lentivirus were purchased from Shanghai Gene Chem Company. Stable-overexpression-MORC2, stable-shRNA-MORC2 and control cell lines were selected with puromycin (2 μg/ml) after infection by lentivirus.

### Cell cycle analysis, growth curve and colony formation assay

Stable overexpressed cell lines were seeded in 60-mm plates to perform the flow cytometry and cell counts assays as described in our previous paper [11, 24]. For colony formation assay, 500 cells were plated in six-well plates to assess the proliferation potential of cells and incubated at 37°C in a 5% CO_2_ incubator. After 2 weeks, the number of colonies was counted. Data represent the mean ± SD from 3 independent experiments performed in triplicate wells.

### Tumorigenicity assay

6-week-old female athymic nude mice were subcutaneously injected with 2 × 10^6^ cells in 0.2 ml PBS into right scapular region. Four groups (5 each) of mice were injected with lentivirus-mediated stable expression of MORC2 (WT, SA and SE) and control vector, respectively. Tumor size was measured every 2 days with calipers. The tumor volume was calculated based on the formula (*L* × *W*^2^)/2, where *L* is length and *W* is width. After the mice were killed at 3 weeks, the weight of the tumors was measured.

### Tissue samples

Samples of human gastric cancer tissues and paired-adjacent non-tumor gastric tissues further than 5 cm from the tumors were obtained from 210 gastric cancer patients who were underwent gastric resection surgery in the 1st hospital of China Medical University. These gastric cancer tissues and adjacent normal tissues of them were performed immunohischemical staining. 68 pairs of fresh samples were snap frozen in liquid nitrogen immediately after resection and stored at –80°C until protein extraction for Western Blot. All samples were obtained with patients' informed consent.

The samples were histologically confirmed by staining with hematoxylin-eosin. The histological grade of cancers was assessed according to criteria set by the World Health Organization.

### Immunohistochemistry

Paraffin-embedded gastric tumor tissues were obtained from the First Hospital of China Medical University. Five-micrometer-thick consecutive sections were cut and mounted on glass slides. The slides were deparaffinized, and rehydrated, prior to antigen retrieval, and blocking endogenous peroxidases. The sections were then washed three times in 0.01 mol/L PBS for 5 minutes each and blocked for 1 h in 5% normal goat serum. The sections were exposed to anti-phosphor-MORC2 Ser677 (1:100) and anti-PAK1 (1:200) 4°C overnight. After brief washes in 0.01 mol/L PBS, sections were exposed for 2 h to 0.01 mol/L PBS containing horseradish peroxidase-conjugated goat anti-rabbit immunoglobulin G (1:200), followed by development with 0.003% H_2_O_2_ and 0.03% 3, 3′-diaminobenzidine in 0.05 mol/L Tris-HCl.

All of the immunostained sections were reviewed by two authors who had no knowledge of the patients' clinical status. Five areas selected at random were scored. All sections were scored in a semiquantitative manner according to a previously described method, which reflects both the intensity and percentage of cells staining at each intensity [26]. Intensity was classified as 0 (no staining), +1 (weak staining), +2 (distinct staining), or +3 (very strong staining). A value designated as the ‘HSCORE’ was obtained for each slide by using the following algorithm: HSCORE = ∑ (I × PC), where I and PC represent the staining intensity and the percentage of cells that stain at each intensity, respectively. And the corresponding HSCOREs were calculated separately. The results were evaluated separately by 2 independent observers. Immunohistochemical results were judged by HSCORE [27, 28] (histological score).

To test for the specificity of the phospho-MORC2 Ser677 antibody, the antibody was preincubated for 1 h at room temperature with the peptide used for immunization at a mass ratio of 1:5, prior to application to the tissue slides.

### Statistical analysis

All statistical analyses were carried out using the SPSS 16.0 software and the results were considered to be significant when the *P* value was < 0.05. Data are presented as mean ± SD from at least three separate experiments. Statistical analysis was performed with Student's *t*-test, non-parametric test (Mann–Whitney *U* test between 2 groups and Kruskall–Wallis test for 3 or more groups). The statistical significance of correlations was calculated by a chi-square test and Spearman's rank correlation.

## SUPPLEMENTARY MATERIALS AND METHODS


