# Supplementary material for: PAK1-mediated MORC2 phosphorylation promotes gastric tumorigenesis
Source: Oncotarget. 2015 Mar 21;6(12):9877–86. doi: 10.18632/oncotarget.3185 (PMC4496403; doi:10.18632/oncotarget.3185)
Supplement: Supplementary file 1 [file oncotarget-06-9877-s001.pdf]

SUPPLEMENTARY MATERIALS AND METHODS

GST pull-down assay

For *in vitro* GST pull-down, *In vitro* transcription and translation of the PAK1 and MORC2 proteins were performed by using the TNT-coupled transcription-translation system (Promega). The GST pull-down assays were performed by incubating equal amounts of GST, GST-PAK1, and GST-MORC2 fusion proteins immobilized on GST Sepharose beads (Amersham Biosciences) with *in vitro* translated ^{35}S-labelled PAK1 or MORC2 protein. The bound proteins were then visualized by autoradiography. Crosslinking analysis of PAK1, purified GST-PAK1 was incubated with an increasing amount of *in vitro* translated ^{35}S-labelled MORC2 and further crosslinked using a zero-length crosslinker EDC/NHC at 4ºC for 2 hrs. Crosslinked products were denatured by heating in 3 x SDS buffer and separated on an SDS-PAGE gel followed by autoradiography.

PAK1 kinase assay

Using myelin basic protein (MBP) or GST-MORC2 fusion protein, we performed *in vitro* kinase assays in HEPES buffer (50 mM HEPES, 10 mM $MgCl_2$, 2 mM $MnCl_2$, 1mM dithiothreitol) containing 100 ng of purified GST-PAK1 kinase (cell signaling), 10 µCi of [γ-^{32}P] ATP, and 25 µM cold ATP. GST proteins were purified by using glutathione Sepharose (Amersham Biosciences) according to the manufacture's instructions. The reaction was carried out in a volume of 30 µl for 45 min at 30ºC and then stopped by the addition of 6 µl of 6 x SDS loading buffer. We analyzed the reaction products by SDS-PAGE and autoradiographed them. The purified GST-MORC2 was then incubated with PAK1 kinase in HEPES buffer containing 1 µl of γATP in a final reaction volume of 30 µl.

SUPPLEMENTARY FIGURE

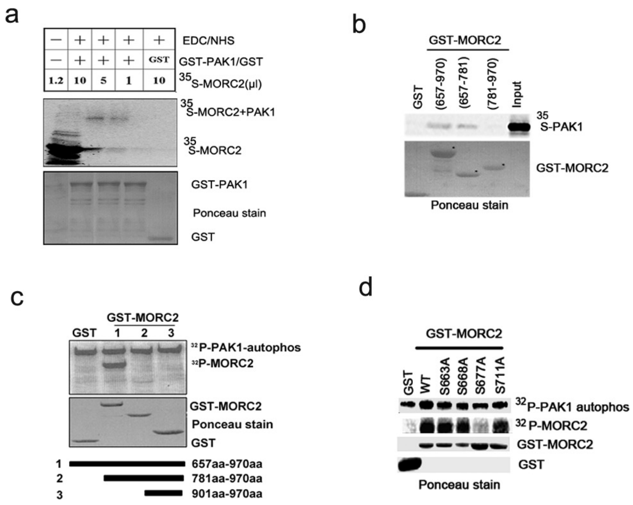

Supplemaetary Figure S1: (a–b) MORC2 interacts with PAK1 *in vitro*. (a) Crosslinking experiments used the zero-length crosslinker EDC/NHS conjugate two interacted protein of dose-reduced *in vitro* translated ^{35}S–MORC2 (657–781) and GST-PAK1. (b) *In vitro*–transcribed and translated ^{35}S–PAK1 binds to GST-MORC2 deletions. For *in vitro* GST pull-down assay, GST, and GST-MORC2 deletions fusion proteins were incubated with ^{35}S- labeled PAK1 proteins transcripted and translated *in vitro*. Bound proteins were detected with ^{35}S-labeled PAK1 autoradiography. Black stars indicated the GST-fusion proteins. **(c–d)** MORC2 is phosphorylated by PAK1 on Ser677 *in vitro* kinase assay. (c) PAK1 phosphorylates the regions of GST-MORC2 (657 – 781) constructs *in vitro*. (d) PAK1 phosphorylates MORC2 on Ser 677 *in vitro*. Mutations of MORC2 from serine to alanine between amino acids 657 and 781 were performed in PAK1 kinase assay.
